# Photopolymerization Inhibition Dynamics for Sub-Diffraction Direct Laser Writing Lithography

**DOI:** 10.1002/cphc.201200006

**Published:** 2012-03-05

**Authors:** Benjamin Harke, Paolo Bianchini, Fernando Brandi, Alberto Diaspro

**Affiliations:** a Department of Nanophysics, Istituto Italiano di Tecnologia, Via Morego 3016163 Genova (Italy), Fax: (+39) 010-71-781-762; b Department of Physics, University of Genova16146 Genova (Italy)

**Keywords:** lithography, polymerization, resolution, sub-diffraction, triplet state

The significance of direct laser writing (DLW) lithography increased considerably during the last years.^1^–^3^ The ability of writing arbitrary structures in all directions into a liquid resin opened up new applications, such as photonic crystal devices^4^ or cell scaffolding.^5^ Especially, creating structures along the optical axis—that is, inside the resin—shows a major advantage in comparison to conventional electron beam or mask lithography. The mechanism on which DLW is based is free radical polymerization. Hereby, a photoinitiator is excited by one or two-photon excitation (1PE and 2PE, respectively) into the first excited singlet state. Since typically photoinitiators are now designed to show a very high intersystem crossing (ISC) rate (>85 %) the excited molecule will populate its triplet state from where it can react with environmental molecules forming a radical compound that can start the polymerization reaction of the resin, which is mostly an acrylate. By carefully choosing the resin^6^ and the photoinitiator, very solid structures with sub-micron sizes can be fabricated. The latter can even be further reduced by the presence of a strongly defined threshold for polymerization mainly due to free oxygen, which reacts with free radicals before they can initiate the polymerization. In fact, a certain concentration of excited initiator molecules must be generated so that a sufficient number of radicals can start a cross-linking reaction. Thus, tuning the excitation intensity close to that threshold leads to a volume of polymerization with an extension in the order of the diffraction-limited size of the light spot. Moving to 2PE of the photoinitiator introduces a non-linearity that even enhances the threshold effect.

The presence of a strictly defined threshold can, in theory and experiment, lead to sub-diffraction feature sizes,^7^ when the excitation power is tuned so that a sufficient amount of radicals for polymerization is generated only in the center of the excitation spot. However, experimentally it is challenging to stably set the excitation power so close to the threshold since it can depend on temperature, substrate, chemical environment and concentration fluctuations. So, all in all, to achieve sub-diffraction resolution, the method is sensitive to “normal” experimental noise. Based on a concept published several years ago,^8^ a very recent approach^6^, ^9^–^13^ to reduce the feature size in DLW lithography was inspired by the STED (stimulated emission depletion) microscopy technique.^14^–^16^ Hereby, the diffraction-limited excitation spot is overlaid by a second beam red-shifted with respect to the excitation-beam wavelength. By stimulated emission depletion, this second beam switches off the excited molecules before they can emit a fluorescence photon. Featuring zero intensity in the center of this STED beam, as for example with a doughnut-shaped beam, leaves the molecules in the excitation maximum untouched. An effective fluorescent area of sub-diffraction size is achieved by saturating the switching process so that also molecules located closer to the center of the excitation beam are switched off. This fact allows having theoretically unlimited resolution, which is of major importance: once this technique can be applied in lithography, the resolution is not just shifted to another limit (like reducing the excitation wavelength to the UV) but can be enhanced to an arbitrarily high value. The STED technique is based on reversible saturable optical fluorescence transition (RESOLFT),^17^ which is a more generalized concept allowing the switching states to be completely arbitrary (fluorescence, spin,^18^ etc). The theoretical description of its resolution *d* can be given by Equation (1):^19^, ^20^

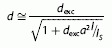
(1)

where *d*_exc_ defines the diffraction-limited size of the excitation focus, which depends on its wavelength and the numerical aperture of the imaging system. *a* is a system constant representing the shape of the switching-beam focus. The applied inhibition beam intensity *I* normalized to its saturation intensity *I*_S_—which defines the intensity required to switch off half of the excited molecules—governs the resolution of the optical system. The fraction *I*/*I*_S_ has to be raised up in order to achieve a sub-diffraction resolution. This can be done by either increasing the inhibition beam intensity *I* or optimizing the cross-section for the switching process to reduce *I*_S_. In DLW lithography, the RESOLFT concept can be used to reduce feature sizes by selective inhibition of the polymerization reaction. Here, the switching beam has to interact with the photoinitiator molecules to stop the generation of radicals and thereby inhibit the polymerization reaction. However, to perform high-resolution DLW lithography with best performance, a detailed understanding of the process to inhibit polymerization is of great importance.

Herein, we present photopolymerization experiments based on a regular STED setup that is also used to perform high-resolution lithography experiments. We concentrate here on the polymerization inhibition dynamics illustrating the possible photophysical pathways using 1PE and 2PE. Measurements focused on the time dynamics of the inhibition process identify the responsible mechanism to be based on a long-living state like the triplet state.

The setup used to perform the experiments is a standard STED setup whose basic geometry has been previously published.^19^ A coarse schematic is shown in Figure 1 a. Four laser lines—405 nm (CW, Cube, Coherent, USA), 532 nm (CW, FiberTec, AMS Technologies, Germany), 642 nm (CW, MPB communications, Canada), and 740–760 nm (pulsed, 76 MHz, ∼150 fs pulse length, Chameleon Vision, Coherent, USA)—are overlaid and focused onto the sample by an oil-immersion objective lens (OL, NA 1.4, Leica Microsystems, Germany). A piezo-controlled translation stage (Nano LP100, Mad City Labs, USA) performs the scanning by moving the sample. The 532 nm and 642 nm laser lines are used for CW excitation and CW STED depletion, respectively. Herewith standard STED measurements are performed to routinely check the functionality of the optical setup. Example measurements on a fluorescent beads sample (Nile red, 40 nm, Invitrogen, USA) are shown in Figure 1 b.

**Figure 1:**
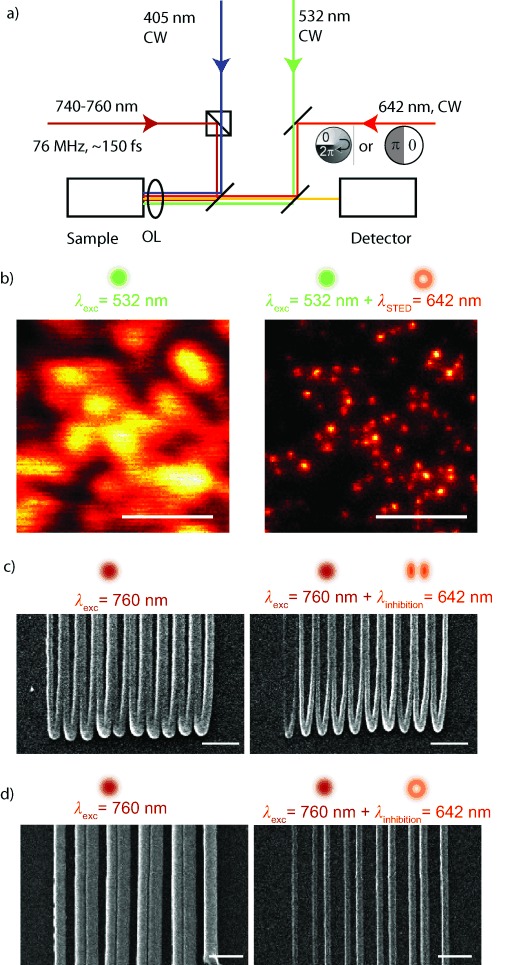
a) Schematic of the optical setup used to perform high-resolution STED and lithography experiments. For routinely checking the performance of the microscope, high-resolution imaging was performed on fluorescent spheres (40 nm, nile red, Invitrogen, USA) b) Left: confocal image, excitation at 532 nm. Right: STED counterpart featuring nearly all the details of the sample, depletion at 642 nm. Raw data are presented. SEM images of high-resolution lithography experiments based on the photoinitiator ITX are shown in (c) and (d). For two different phase plate geometries [in (c) a 0–*π* phase jump, in (d) a 0–2*π* vortex shape], a clear increase in contrast and resolution can be observed. Scale bars: 1 μm. Parameters in (b): pixel size 20 nm, pixel dwell time 150 μs, STED power 590 mW. Parameters in (c): writing speed 0.1 μm ms^−1^, 2PE power *P*_2PE_=8.0 mW, inhibition power *P*_*i*nh_=33.2 mW. Parameters in (d): writing speed 0.1 μm ms^−1^, *P*_2PE_=11.5 mW, *P*_inh_=43.0 mW. All power values are measured in the back aperture of the objective lens (OL).

For polymerization measurements, 1PE is realized by the 405 nm CW line and 2PE by the near-infrared line at 740–760 nm.

The material systems for the polymerization experiments is a pentaerythritol triacrylate (Sigma Aldrich) mixed with 1.5 wt % of the photoinitiator isopropyl thioxanthone (ITX, Sigma Aldrich) or with 0.25 wt % 7-diethylamino-3-thenoylcoumarin (DETC, Exciton, USA). The same material systems have been previously studied,^2^, ^11^ and it has been shown that high-resolution lithography can be achieved. For the sample preparation, we drop-casted ∼100 μL of the resin on a regular glass cover slip. After exposure, we washed the sample about 5 min in methanol followed by another 5 min washing in isopropanol. We performed first experiments on high-resolution lithography with the presented setup based on the photoinitiator ITX. Scanning electron microscope (SEM) images are presented in Figure 1 c,d, in which we used two different phase plates within the inhibition beam to test the performance of the system. First, we overlaid the diffraction-limited 2PE spot with an inhibition beam shaped in a way that two maxima are formed along one lateral direction (Figure 1 c). The shape is obtained by retarding half of the back aperture of the OL by *π*.^21^ Here, the polarization of all beams is linear along the direction of the gap between the two maxima. This inhibition pattern should be optimal for achieving small line widths when the writing direction is along the latter gap since any possible interaction of the inhibition beam with already illuminated parts of the sample will have a minor effect. Additionally, we changed the inhibition pattern by replacing the previous phase plate by a helical vortex geometry (VPP-1A, RPC photonics, USA) forming a doughnut-shaped intensity distribution in the focus (Figure 1 d). This inhibition pattern is mostly used in STED microscopy since it enhances the resolution uniformly within the lateral plane.^19^ For both phase plates used herein, an increase in contrast and resolution is clearly visible and is comparable to already published results.^2^, ^10^ Notably, within the same material system, we observed polymerization inhibition by applying a laser light of 642 nm instead of a 532 nm laser as described in the mentioned literature. This fact is pointing at a broad absorption spectrum of the contributing photoinitiator molecule giving already some hints for the possible photophysical pathways responsible for the polymerization inhibition effect. To explore these pathways more in detail, we performed polymerization inhibition experiments focused on the time dynamics of the inhibition process. For these measurements, one has to consider the fact that usual photoinitiators have a very low fluorescent quantum yield with respect to any common dye molecules used in microscopy. Even if ITX shows a comparable high value of ∼15 %,^10^ the fluorescence signal during a polymerization experiment is too low to be analyzed. For analyzing a fluorescence signal, the pure photoinitiator has to be dissolved in a solvent without an acrylate. These studies have been previously reported in the literature.^22^ However, changing the chemical environment of the photo initiator can change all the dynamics of such a system. To get insights about the time properties of the initiator in its final chemical environment, we performed time-resolved experiments by investigating the performance of the polymerization inhibition for different timing settings. For these studies, 1PE was firstly used to avoid any side effects due to the non-linear absorption. We modulated at a frequency of 100 kHz and a pulse width of 100 ns the 1PE and the 642 nm inhibition laser, (Figure 2 a). We used an acousto-optic modulator (AOM, AA Optoelectronic, France) to modulate the 642 nm laser line, while the intensity of the 405 nm laser was directly controlled by applying a modulation voltage to the laser controller. A time delay Δ*t* between the two pulses can be electronically applied by a two-channel pulse generator (Agilent, USA). For all the following experiments, we removed any phase plate in the inhibition beam path to obtain a Gaussian beam profile in the focal area. Figure 2 b shows the polymerization inhibition for different time delays. Therefore, a single line was written in which the inhibition laser was sequentially turned on and off to observe the effect of the polymerization inhibition. When the modulation frequency applied to the two laser lines overlays in time (Δ*t*=0), the polymerization can be effectively inhibited, which would be the same behavior as that observed for beams without intensity modulation. For a phase shift of Δ*t*=280 ns, the partial inhibition can be measured, which means that the photoinitiator (to some extent) either already forms a radical or already causes cross-linking of the acrylate before the inhibition laser deactivates the system. For Δ*t*=1930 ns, nearly no inhibition can be observed, which is due to the complete generation of radicals or cross-linked material within this time range. Importantly, also no effect on the polymerized line can be observed for a negative time shift (Δ*t*<0). Here, the inhibition pulse arrives when the photoinitiator molecules are not excited yet and no effect of this inhibition light can be observed. This is a direct proof that the inhibition light interacts with an excited photoinitiator molecule and not with the acrylate itself. For a quantitative analysis of these measurements, we used SEM images to investigate how efficient the polymerization inhibition process worked under the given timing conditions. Therefore, the contrast of regions with and without the presence of the inhibition beam was measured and plotted by the black data points in Figure 2 c for ITX and in Figure 2 d for DETC. The inhibition efficiency drops down to 50 % after ∼800 ns for ITX and after ∼5 μs for DETC. In STED microscopy, the inhibition effect is based on the quenching of the fluorescence signal that is not measureable during a polymerization experiment. Therefore, we dissolved the photoinitiator molecules purely in ethanol and measured the emitted fluorescent signal within the wavelength band 520–560 nm. The red graphs display the STED effect for different time delays. As expected from the STED theory, once the pulses of the excitation and inhibition laser show an overlap, a STED effect (even though weak) can be observed for both photoinitiators. However, the STED effect is based on the fluorescent S_1_ state^23^ with a typical lifetime of several ns. Once the pulses of excitation and inhibition beam do not overlap anymore (means Δ*t*>100 ns), no STED effect in each of the photoinitiators is visible. These measurements demonstrate that under the given experimental conditions, photophysical interactions based on the fluorescent state, like STED, play a minor role for polymerization inhibition in the two different photoinitiators.

**Figure 2:**
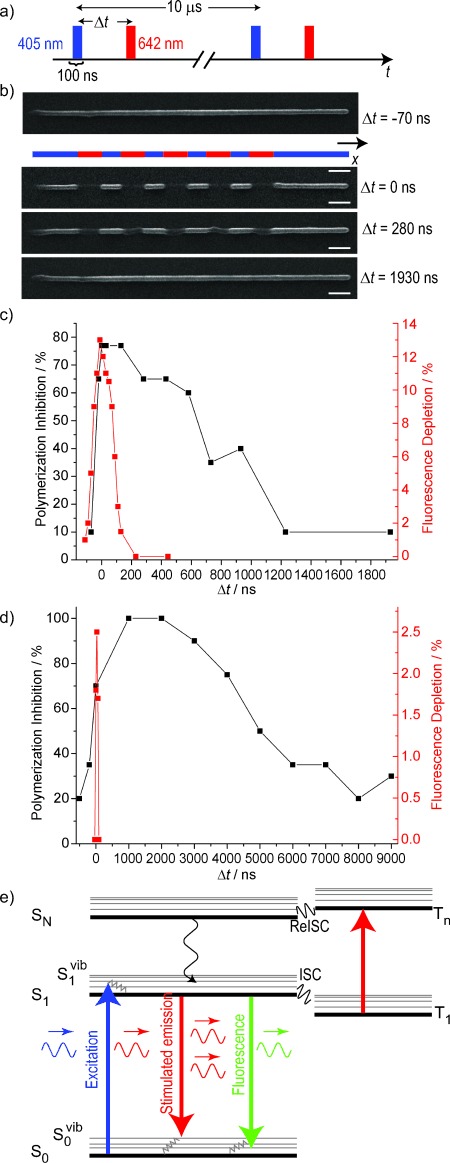
a) For the timing experiments, the 405 and 642 nm lasers were intensity-modulated by a squared signal with a fixed frequency and pulse width of 100 kHz and 100 ns, respectively. b) During a line scan, the inhibition laser was sequentially switched to investigate the timescales of the polymerization inhibition. SEM micrographs of polymerized lines using ITX are presented for different timing settings of the excitation and inhibition laser. Scale bars: 1 μm. c,d) Quantitative analysis of the before-mentioned measurements shows the timing behavior of the inhibition process for the two different photoinitiators ITX (c) and DETC (d). Writing parameters (average powers in the back aperture of the objective lens) for ITX: *P*_1PE_=0.4 μW, *P*_*i*nh_=280 μW; for DETC: *P*_1PE_=1 μW, *P*_inh_=550 μW inhibition power. The STED effect was measured for both photoinitiators in ethanol solution by the reduction of the fluorescence signal and is presented in the red graph (right *y* axis). e) Simplified scheme of the Jablonsky diagram showing the energy levels of the photoinitiator molecules possibly involved in the inhibition process.

Since the timescale of this inhibition effect is within the same range as a typical triplet liftetime, a mechanism based on the triplet state is probable. The singlet–triplet transition is a forbidden pathway and therefore a molecule populating the triplet state may not be stimulated directly to its singlet ground state. One possible pathway to deplete the triplet state population and recover the molecule back to the singlet state is known as reverse inter-system crossing (ReISC).^24^, ^25^ The simplified Jablonsky diagram of the possible energy levels of the photoinitiator molecule is shown in Figure 2 e. Once the molecule is excited to the singlet state S_1_, it can undergo an inter system crossing (ISC) event populating the triplet state T_1_ from where usually a radical is formed. The inhibition laser is now able to excite the molecule to an excited triplet state T_*N*_ from which an ReISC transition to an excited singlet state S_*N*_ is favored. By internal conversion it can relax non-radiatively to the first excited singlet state S_1_, which was the initial state of this process sequence. The effect of ReISC has been demonstrated especially in dye molecules with a high ISC rate, which is also the case for ITX (ISC rate ∼85 %) and DETC (∼97 %) used here. The T_1_–T_*N*_ absorption of ITX was previously investigated by laser flash photolysis.^26^, ^27^ It has been found that the triplet state shows a significant absorption in the wavelength around 600–650 nm, that is, the same range as our inhibition laser. Also, the reported triplet lifetimes in the presence of an acrylate are in the range of 1–10 μs,^26^ matching our experimental results. For the RESOLFT concept, the long life time of the triplet system with respect to the singlet-state lifetime is even favorable: in STED microscopy, high intensities in the inhibition beam are required to effectively reduce the fluorescence because the STED pathway has to compete with the fluorescence transition rate *k*_fl_*=*1/*τ*_fl_ with a lifetime *τ*_fl_ of a couple of ns. The triplet lifetime, which is two to three orders of magnitude longer, allows much lower intensities to achieve the same inhibition rate, enabling also the use of CW lasers with comparable low intensities as we did here. Recently, a work based on a photoswitchable protein showed sub-diffraction resolution based on the RESOLFT concept by using considerable low intensities due to the long lifetime of the involved states.^28^

The triplet state is prone to be the initial state for the polymerization reaction. In microscopy, the triplet state is known to be responsible for most of the photobleaching pathways.^20^, ^29^ The bleaching rates for dyes used in microscopy depend on the use of 1PE or 2PE.^30^ To see whether, also for photopolymerization, the states involved for the radical generation change, we performed time-resolved experiments using 2PE. Here, the laser line generated by the ultrafast pulsed laser was modulated by an AOM and in this way a burst of pulses with a controllable length could be generated. We performed the timing experiment in the same way as for the case of 1PE and the results are plotted in Figure 3. Note, the burst length for the excitation and inhibition laser was set to 100 ns in the case of DETC and 200 ns in the case of ITX due to its lower 2PE cross-section. The timing behavior for polymerization inhibition changes significantly and cannot be determined precisely since the lifetime of the state generating the reacting radicals seems to be shorter than the burst length. This effect can be explained due to absorption of the 2PE light causing also a T_1_–T_*N*_ transition, which has been observed in 2PE microscopy^30^, ^31^ This triplet absorption adds another pathway to depopulate the T_1_ state and therefore results in a significant reduction of the T_1_ lifetime and so in a fastening of the polymerization inhibition dynamics.

**Figure 3:**
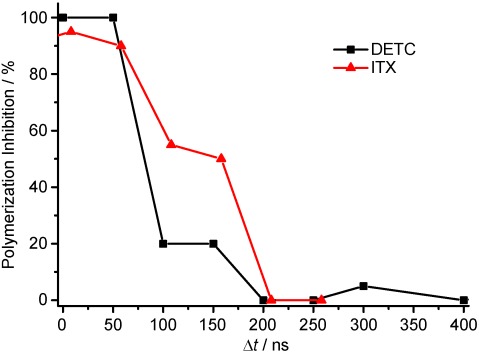
Polymerization inhibition dynamics for 2PE for the two photoinitiators at a wavelength of 740 nm. Note that the burst length for the ITX experiments was set to 200 ns whereas in the experiments with DETC, it was set to 100 ns. Writing parameters (average powers in the back aperture of the objective lens) for DETC: *P*_2PE_=250 μW at *7*40 nm, *P*_inh_=2,75 mW; for ITX: *P*_2PE_=63 μW at 740 nm, *P*_inh_=1,375 mW.

To make sure that the observed effects are based on an interaction of the photoinitiator molecule with the incident light, and not due to light absorption of the acrylate or any other molecules within the compound, we performed qualitative experiments, focused on the ability of inhibiting polymerization, with another acrylate (acrylate II: trimethylolpropane ethoxylate triacrylate, Sigma Aldrich) and one further photoinitiator (BAPO: phenylbis(2,4,6-trimethylbenzoyl)phosphine oxide, Sigma Aldrich). The results are listed in Table 1: the previously described photoinitiators ITX and DETC perform very similar also in a different acrylate (acrylate II) while polymerization started by another photoinitiator (BAPO) in the previously described acrylate (acrylate I) cannot be inhibited. This leads to the conclusion that the effect of polymerization inhibition is based on the interaction of the inhibition beam with the excited photoinitiator molecule. The triplet state of the photoinitiator molecule has to show a significant absorption within the inhibition beam wavelength and therefore cannot be replaced by any other molecule.

**Table 1:** Qualitative investigation of polymerization ability (green symbol) and polymerization inhibition ability (red symbol) for different photoinitiator–acrylate combinations. 1PE and 2PE were performed at 405 and 760 nm, respectively.[a]

Acrylate	Initiator	1PE		2PE	
acrylate I	ITX	✓	✓	✓	✓
acrylate I	DETC	✓	✓	✓	✓
acrylate I	BAPO	✓	χ	✓	χ
acrylate II	ITX	✓	✓	✓	✓
acrylate II	DETC	✓	✓	✓	✓

[a] Acrylate I: pentaerythritol triacrylate; acrylate II: trimethylolpropane ethoxylate triacrylate; ITX: isopropyl thioxanthone; DETC: 7-diethylamino-3-thenoylcoumarin; BAPO: phenylbis(2,4,6-trimethylbenzoyl)phosphine oxide.

Herein, we presented timing experiments for investigating the dynamics of polymerization inhibition. Based on a regular STED setup, we performed polymerization experiments in which we analyzed the timescales needed for inhibiting the polymerization. It turned out that the STED effect used in nanoscopy and based on the depletion of the fluorescence signal is not the dominating effect responsible for the inhibition process and plays obviously a minor role. Since (in the case of 1PE) the timescales in which inhibition can be performed are in the same range as the typical lifetime of a triplet state, an effect based on the interaction with this triplet state should be favored. The measurement based on 2PE shows a significant shortening of these timescales, that is, in accordance to a T_1_–T_*N*_ absorption of the excitation light. This effect has been previously observed by enhanced photobleaching in microscopy due to 2PE. These measurements can contribute significantly to the understanding of the comparatively new field of high-resolution lithography. Once the mechanism is fully explored, an optimization of the material system as well as the experimental conditions should lead to lithography results with even higher resolution.

We presented also high-resolution lithography measurements based on a standard STED setup. A clear increase—relative to structures written by 2PE—in both contrast and resolution applying two different inhibition patterns could be pointed out. These measurements encourage the use of multiple phase plates at the same time. Combining a phase plate optimized for increasing the axial resolution in combination with one for the lateral plane^32^ should give a feature size significantly reduced along all spatial directions.^2^ Due to the clear fact of the comparatively low intensities required for the inhibition process, a parallelization of the illumination can be of great interest since it allows structured illumination^33^, ^34^ or multi-spot excitation,^35^, ^36^ resulting in a substantially reduced processing time.

## References

[1] Kawata S, Sun HB, Tanaka T, Takada K (2001). Nature.

[2] Fischer J, Wegener M (2011). Opt. Mater. Express.

[3] Leong TG, Zarafshar AM, Gracias DH (2010). Small.

[4] Von Freymann G, Ledermann A, Thiel M, Staude I, Essig S, Busch K, Wegener M (2010). Adv. Funct. Mater.

[5] Lee SH, Moon JJ, West JL (2008). Biomaterials.

[6] Stocker MP, Li L, Gattass RR, Fourkas JT (2011). Nat. Chem.

[7] Sun HB, Takada K, Kim MS, Lee KS, Kawata S (2003). Appl. Phys. Lett.

[8] Hell SW (2004). Phys. Lett. A.

[9] Li L, Gattass RR, Gershgoren E, Hwang H, Fourkas JT (2009). Science.

[10] Andrew TL, Tsai HY, Menon R (2009). Science.

[11] Fischer J, von Freymann G, Wegener M (2010). Adv. Mater.

[12] Scott TF, Kowalski BA, Sullivan AC, Bowman CN, McLeod RR (2009). Science.

[13] Fourkas JT (2011). Opt. Photonics News.

[14] Hell SW, Wichmann J (1994). Opt. Lett.

[15] Westphal V, Hell SW (2005). Phys. Rev. Lett.

[16] Hell SW (2009). Nat. Methods.

[17] Hofmann M, Eggeling C, Jakobs S, Hell SW (2005). Proc. Natl. Acad. Sci. USA.

[18] Maurer P, Maze J, Stanwix P, Jiang L, Gorshkov A, Zibrov AA, Harke B, Hodges J, Zibrov AS, Yacoby A (2010). Nat. Phys.

[19] Harke B, Keller J, Ullal CK, Westphal V, Schönle A, Hell SW (2008). Opt. Express.

[20] Kasper R, Harke B, Forthmann C, Tinnefeld P, Hell SW, Sauer M (2010). Small.

[21] Westphal V, Kastrup L, Hell SW (2003). Appl. Phys. B.

[22] Wolf TJA, Fischer J, Wegener M, Unterreiner AN (2011). Opt. Lett.

[23] Rittweger E, Rankin B, Westphal V, Hell S (2007). Chem. Phys. Lett.

[24] Redmond RW, Kochevar IE, Krieg M, Smith G, McGimpsey WG (1997). J. Phys. Chem. A.

[25] Ringemann C, Schönle A, Giske A, von Middendorff C, Hell SW, Eggeling C (2008). ChemPhysChem.

[26] Amirzadeh G, Schnabel W (1981). Makromol. Chem.

[27] Aydin M, Arsu N, Yagci Y, Jockusch S, Turro NJ (2005). Macromolecules.

[28] Grotjohann T, Testa I, Leutenegger M, Bock H, Urban NT, Lavoie-Cardinal F, Willig KI, Eggeling C, Jakobs S, Hell SW (2011). Nature.

[29] Donnert G, Eggeling C, Hell SW (2007). Nat. Methods.

[30] Patterson GH, Piston DW (2000). Biophys. J.

[31] Michalet X, Kapanidis AN, Laurence T, Pinaud F, Doose S, Pflughoefft M, Weiss S (2003). Annu. Rev. Biophys. Biomol. Struct.

[32] Harke B, Ullal CK, Keller J, Hell SW (2008). Nano Lett.

[33] Heintzmann R, Jovin TM, Cremer C (2002). J. Opt. Soc. Am. A.

[34] Gustafsson MGL (2005). Proc. Natl. Acad. Sci. USA.

[35] Kato J, Takeyasu N, Adachi Y, Sun HB, Kawata S (2005). Appl. Phys. Lett.

[36] Bingen P, Reuss M, Engelhardt J, Hell SW (2011). Opt. Express.

